# Impact of the COVID-19 Pandemic on the Motor Development of Schoolchildren in Rural and Urban Environments

**DOI:** 10.1155/2022/8937693

**Published:** 2022-05-17

**Authors:** Sara V. Pajek

**Affiliations:** Vodmat Elementary School, Potrčeva ulica 1, 1000 Ljubljana, Slovenia

## Abstract

Social and school closures during the COVID-19 pandemic may have led to significant stagnation in children's motor development, but precise data on this are lacking. We aimed to examine the impact of the pandemic and society closure on motor development of school children and to find differences between rural and urban environments. From the SLOfit database, we obtained anonymous results from 756 6^th^ grade children before the pandemic (11.3 ± 0.5 years, 52.5% boys) who performed physical fitness measurements in 2017 and 2019 in 8th grade and from 853 6^th^ grade children (11.4 ± 0.5 years, 51% boys) who performed measurements in 2019 and 2021, after 3 pandemic waves. The results of eight physical activity tests and the overall physical fitness index were compared between the prepandemic and the pandemic generation. We divided the sample into four groups (rural and urban prepandemic group and rural and urban pandemic group) and compared the changes in test scores between 6th and 8th grade. We found a statistically significant decrease in the physical fitness index of the pandemic generation (from 51.6 ± 29.6 to 45.8 ± 30.3) compared to the prepandemic generation (from 50.4 ± 30.5 to 50.5 ± 29.7), *p* < 0.001. The greatest effects of pandemic closure were found in the 600-meter run, in polygon course backwards test, in the number of sit-ups in 60 seconds, and in the 60-meter sprint. Children from rural areas showed worse decrement in physical fitness index compared to urban areas, except for 600-meter run. We conclude that the pandemic closure has had a significant inhibitory effect on the motor development of schoolchildren and has reduced their overall physical fitness with worse decline in rural areas. The pandemic generation of children needs more physical education in schools and other systemic interventions to mitigate these consequences.

## 1. Introduction

In the Republic of Slovenia, the COVID-19 pandemic was declared on March 12, 2020 [1]. Since the beginning of the pandemic, we have been hearing warnings from experts that the pandemic-related closure of schools and sports facilities, as well as the restriction of outdoor exercise, will have a negative impact on the physical (and mental) condition of the population, including children [2, 3]. Pandemic measures, including the closure of schools and kindergartens, have eliminated regular physical education, which accounts for a large part of planned physical activity of schoolchildren [4]. Accordingly, the first reports on the impact of the pandemic closure showed a decline in children's physical fitness after the first wave of the pandemic in 2020 [5]. The first wave was followed by subsequent waves, and therefore there is a need for reliable data on the impact of the protracted pandemic course on the motor development of children in Slovenia.

The Sports Educational Chart (Slovenian: »športno-vzgojni karton«) and its upgrade, the SLOfit project, is the main system used in Slovenia to monitor and assess the physical and motor development of school children [6]. In this system, motor development has been assessed in all Slovenian primary and secondary schools since 1987. The results are evaluated by the Faculty of Sport at the University of Ljubljana, Laboratory for Diagnostics of Physical and Motor Development [7]. Before the pandemic, from 2010 to 2019, a trend of gradual improvement in physical fitness indicators of schoolchildren was observed in Slovenia [1]. In the first pandemic year 2020, lower, moderate, and high-intensity physical activity as measured by School Health Action, Planning, and Evaluation System (SHAPES) questionnaire was found in a relatively small sample of 62 schoolchildren, but no significant effect on fitness indicators as captured by Sports Educational Chart was found compared with the prepandemic control sample [8].

There are some hereditary and maternal factors associated with early motor development of children [9]. Later in development, parental beliefs and behaviours but also some school and sports environment features such as peer relations, classroom age range, in-class interaction, and teacher education relate to better motor performance as well [10]. Although it is known that physical inactivity associates with obesity and poorer motor abilities [11], there is lack of information on the impact of population-level physical activity on motor development in children. After three pandemic waves, regular measurements of physical fitness were conducted in schools from April 12, 2021, as part of the SLOfit project. This gave us a unique opportunity to analyze the impact of the protracted course of the pandemic in several waves and subsequent society closures with reduction of physical activity on children's motor development.

We designed this research with the main aim to determine the impact of the COVID-19 pandemic on physical fitness and motor development of primary school children. Our secondary aim was to find possible differences between rural and urban areas. On the basis of previous findings of declines in aerobic fitness in a sample of healthy American children during the first pandemic wave [5] and the reported decline in physical activity in Slovenia [8], we expect to find a statistically significantly lower progress of physical performance of the children in pandemic generation (first hypothesis). The pandemic measures are unlikely to have affected people equally in all regions. In densely populated urban areas, children had fewer opportunities to unrestrictedly play outdoors, because of the mandated wearing of masks when interpersonal distance was less than 2 meters and a ban on the use of children's playgrounds. Children in less populated rural communities had an easier access to outdoor places where they could be physically active without restriction. Therefore, we hypothesized that the motor progress of children from urban, densely populated areas in physical performance would be statistically significantly lower than the progress of children from rural, sparsely populated areas (second hypothesis).

## 2. Materials and Methods

We designed this research as a prospective observational study, where we analyzed the results of physical fitness of two generations of schoolchildren: prepandemic generation, which was measured in 2017 in the 6th grade of primary school and in 2019 in the 8th grade, and pandemic generation, which was measured in 2019 in 6th grade and in 2021 in 8th grade of elementary school. In its motor development, the pandemic generation has been exposed to a risk factor—the impact of the pandemic with all its social and health consequences. A brief summary of the key effects of the pandemic and the social distancing and closure measures taken is shown in Table 1. The prepandemic generation was not exposed to these measures and had 3 hours of physical education per week in the 6th grade and 2 hours of physical education per week in the 7th and 8th grade of elementary school. Youth club sports and recreational activities during this period were normal, but during the pandemic period, they were subjected to restrictions and closures.

Since the measurements of physical fitness are performed in the vast majority of Slovenian schools (more than 94% of children in Slovenian public schools), we were able to evaluate possible regional differences in addition to the general impact on motor development. To compare the impact of the COVID-19 pandemic on children from urban and rural areas, we selected participants from the municipalities with the highest and lowest population density in the Republic of Slovenia. Data on population density were taken from the publicly available SiStat database of the Statistical Office of the Republic of Slovenia as of December 21, 2021. We used the table “Population Density and Femininity Index, Municipalities, Slovenia, Semiannual” (Table ID: 05C4010S), which is publicly available. We included schools from the municipalities with the highest and lowest population density and evenly geographically distributed across the Slovenian regions. About 800 children were included in each group. In this way, we included 11 schools from the 18 most densely populated municipalities and, on the other hand, 18 schools from the 27 least populated municipalities (Supplement Table S1 shows all the included municipalities and schools).

All children who participated in the SLOfit measurements in the selected schools and whose parents gave informed consent to participate in the The Sports Educational Chart (the part of SLOfit system that measures schoolchildren in schools) were included in the study sample. Affirmative verbal consent was obtained from all children before each measurement and data collection. Participation was completely voluntary. Data were collected and analyzed anonymously. The collection and anonymous use of data in the SLofit system was approved by the Medical Ethics Committee of the Republic of Slovenia (document ID 102/03/15).

In our study we used the results of SLOfit physical fitness measurements, which were measured every year in April at public elementary schools. The measurement system is described in detail elsewhere [7] and, in addition to measuring height, weight, and triceps skinfolds, includes a battery of fitness tests. This consists of the performance of eight tasks in four groups: (i) 60 second sit-ups, bent arm hang (indicators of muscular fitness), (ii) stand and reach (indicator of flexibility), (iii) standing long jump, polygon course backwards, 20 seconds arm plate tapping, 60 m sprint run (indicators of skills related fitness), and (iv) 600 m run (indicator of cardiorespiratory endurance). The exact description and measurement properties of these tests are given in ref. [6] and ref. [7].

The physical fitness index (XT) was also calculated to represent the average performance at abovementioned 8 motor tests. It is an average value of eight T-scores. The *T*-score is a value that tells us where within the population of children of the same sex the individual's score lies. The *T*-score is calculated by converting the individual's test result to the *Z*-score, multiplying by 10 and adding 50 (quantile normalization) [6].

Descriptive statistics were obtained by calculating averages and standard deviations as all variables were normally distributed. Differences between generations were tested by repeated measures ANOVA. The effect size was calculated in the analysis of variance using the eta squared parameter (*η*^2^). The difference between the 2 generations in 8th grade was calculated as an adjusted difference that included the baseline scores in 6th grade as a covariate (analysis of covariance). The difference between the generations was also expressed in a standardized form by standardizing the calculated adjusted difference between the generations to the overall standard deviation (Cohen's d). Calculated differences in results from 8^th^ and 6^th^ grade were used compared in four groups of children (prepandemic and pandemic urban and rural group) using a test of variance between these four different groups. IBM SPSS Statistics 22 and 27 (IBM corp., NY, USA) were used for the analyses. The limit of statistical significance was always *p* < 0.050.

## 3. Results

### 3.1. Sample Description

The study included 1609 male and female students, 756 of whom belonged to the prepandemic generation, attending 6th grade of elementary school in 2016/2017. The pandemic generation in our sample includes 853 children who entered 6th grade in the 2018/2019 school year and were then exposed to SARS-CoV-2 virus containment measures in 7th and 8th grade. Baseline demographic and anthropometric data for the included children are shown in Table 2.

The same children were included in 6th and 8th grade. Data are given as mean ± SD.

### 3.2. Differences between Prepandemic and Pandemic Generation

The absolute values of the SLOfit fitness test results are shown in Table 3. Columns 3 and 4 show the absolute results for the two generations in 6th and 8th grade, and the *p* value in column 5 was calculated using repeated measures ANOVA for time x group interaction. There was a statistically significant difference between the two generations over time at all tests except for the 20 s arm plate tapping. The adjusted mean difference between the generations in the 8th grade showed similar results—significant differences with poorer results for pandemic generation at all tests except for one. The effect size of time x generation differences, expressed by partial eta squared, was the greatest in the physical fitness index, 600 m run, polygon course backwards, bent arm hang, and 60 m sprint.

**Figure 1:**
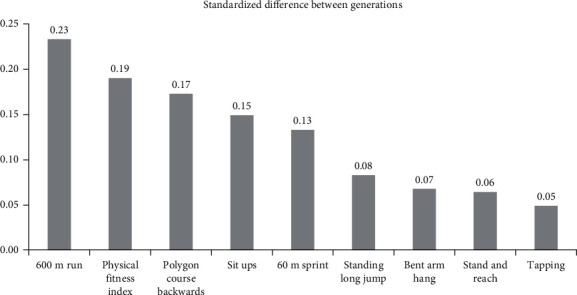
Standardized difference between the generations in 8^th^ grade.

Figure 1 shows the magnitude of the standardized difference in test scores between the prepandemic generation and the postpandemic generation in eighth grade. Standardized differences were determined by dividing the adjusted difference between generations in 8th grade (Table 3, column 6) by the total standard deviation in each test. The results allow for comparison across tests. When we compare these standardized differences, we see that the largest adjusted differences are visible in the 600-meter run, the physical fitness index, the polygon course backwards, the sit ups, and the 60-m sprint. For these tests, we also analyzed the differences between students from urban and rural areas.

### 3.3. Differences between Rural and Urban Areas

Students were divided into four groups according to generation (prepandemic and pandemic) and whether the school was located in an urban or rural setting. Their demographic composition and baseline physical characteristics in 8th grade are shown in Table 4.

BMI: Body mass index.

We can see that the composition by sex and physical characteristics was very similar between the groups. The differences in physical characteristics between groups within each sex were not statistically significant.

**Figure 2:**
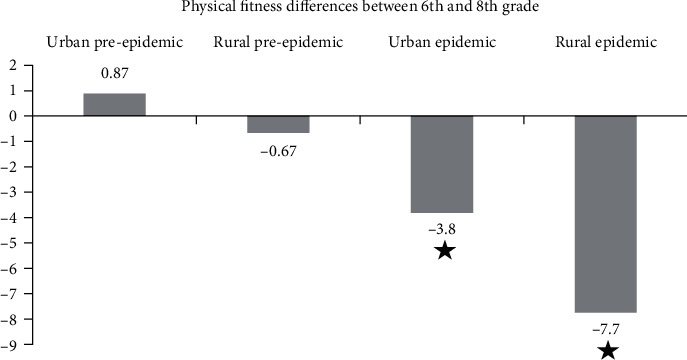
Physical fitness index changes (average) in the four groups.

The physical fitness index as a composite measure of physical fitness shows the largest decrement in rural areas (Figure 2). Stars in Figure 2 designate the significant intragroup differences. The differences between the groups in Figure 2 are statistically significant (*p* < 0.001). The urban pandemic group is significantly different from the urban prepandemic group and the rural pandemic group (*p* ≤ 0.050). The rural pandemic group is significantly different from all other groups (*p* < 0.001 for the comparison with the prepandemic groups and *p* ≤ 0.050 for the comparison with the urban pandemic group).

**Figure 3:**
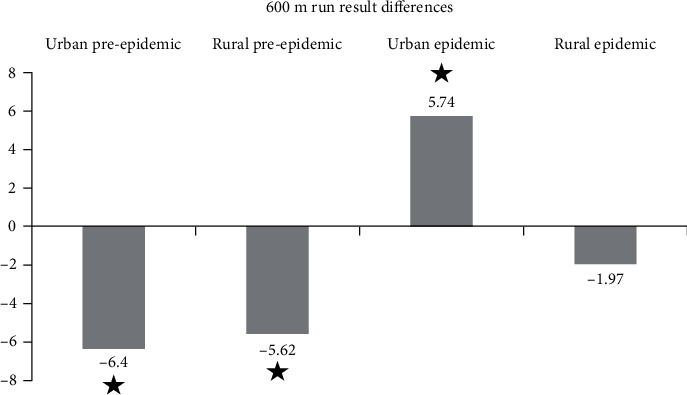
Average difference between the 8^th^ and 6^th^ grade 600 m run time (in seconds).

The urban pandemic group showed the largest deficit in 600 m run with 8^th^ grade results actually being worse than in the 6^th^ grade (Figure 3). In other tests, rural pandemic group was inferior. Differences between the groups for 600 m run in Figure 3 are statistically significant (*p* < 0.001), all differences between urban pandemic and other groups are also significant (*p* < 0.001). The differences between rural pandemic and prepandemic groups are not significant. Stars designate the significant intragroup differences.

## 4. Discussion

Our results show a large and statistically significant impact of the COVID-19 pandemic on the motor development of schoolchildren. When we look at the differences in motor development between the prepandemic generation and the pandemic generation, we find that the pandemic generation had statistically significant lower progress in motor abilities as measured in all fitness tests except for the 20 second arm plate tapping. The effects of the pandemic were the greatest in the 600-meter run, polygon course backward, sit ups, and the 60-meter sprint. The greatest negative effects were seen in the endurance (600-meter run) and skill-related motor tests (60-meter sprint, polygon course backwards), and upper-body muscle fitness (60 second sit-ups). As a result, there was also a large effect on the physical fitness index, which is a composite measure of physical fitness. Based on these results, we can confirm the first hypothesis and conclude that the measured age progress in physical fitness of children exposed to pandemic measures is statistically significantly lower than the progress of children of the same age in the generation before the pandemic.

We could not confirm the second hypothesis that the progress of children from urban, densely populated areas in terms of physical performance would be statistically significantly lower than the progress of children from rural, sparsely populated areas. Conversely, the physical fitness index deteriorated more in the rural pandemic group than in the urban pandemic group, which is the opposite of what we expected. The only fitness test in which children from the urban pandemic group performed largely worse than the others was the 600-meter run, in which scores actually deteriorated significantly between 6th and 8th grade, and the expected progress with age did not occur at all. This confirms the significant effect of the difference in access to the external environment between the urban and rural environments on the maintenance of cardiorespiratory endurance, which was the basis for our second hypothesis. However, the greater decline in the overall index of physical fitness in the rural group of children suggests that overall physical incentives declined more in rural areas than in urban areas at the time of the pandemic.

Comparing our results with recent reports by other investigators, we note that both French [13] and Austrian authors [14] found a decline in fitness in schoolchildren after the first pandemic wave. To our knowledge, our study is the first to report the effects of pandemic over the prolonged duration of three waves and to include a (historical) control group. Chambonniere et al. measured 106 3rd- and 4th-grade elementary school children (aged 9 and 10 years) in February 2020 and 100 additional children at the same grade level in January 2021. They described significant deteriorations in cardiorespiratory endurance, standing long jump, ball throw, and cognitive function [13]. In contrast to our study, they compared the results of different children without a control group. However, Jarnig et al. reported a significant decrease in distance in a 6-minute running test in 764 Austrian children aged 7-10 years and a significant increase in the proportion of overweight and obese children [14]. This study also had no control group. In contrast to the Austrian researchers, we found no significant differences in body mass index between eighth graders from the prepandemic and pandemic generations.

Limitations of our study include the possibility of bias in the selection of schools in the municipalities, since we followed the principle of even geographical representation in selecting municipalities at the high and low ends of the settlement density scale and did not select them randomly. Another limitation is not including the schools from municipalities with a medium settlement density in the Republic of Slovenia. Sensitivity analysis should also be performed with a separate analysis of the results by gender.

## 5. Conclusions

In our study, we showed that COVID-19 pandemic significantly affected the motor development of schoolchildren with major deficits in domains of cardiorespiratory endurance (600 m run), skill-related fitness (polygon course backwards, 60-m run), and core strength (sit-ups). In general, the effects of the pandemic were larger in rural areas. Our research will help physical education teachers and coaches in sports clubs to plan physical activity programs for children. Given the characteristic negative changes in children's motor development, our results justify the need to closely monitor the development of the pandemic generation and take systematic corrective measures. First and obvious corrective measure would be to increase the number of physical education hours for the pandemic-affected generation of children.

## Figures and Tables

**Table 1:** Key societal actions during the pandemic and before the last pandemic generation testing [12].

Pandemic wave (year)	Time frame	Measures taken
The first (spring 2020)	4^th^ March–14^th^ May 2020	Restrictions on gathering in public places and movement in public areas (individual movement allowed) Closure of schools and other educational institutions∗ Closure of public transportation Closure of stores and services, including sports clubs (with exceptions, e.g., grocery stores, pharmacies, and gas stations) Prohibition of movement outside the residential community Mandatory use of a protective mask in enclosed public spaces
The second (autumn 2020 and winter 2020/21)	4^th^ September–8^th^ February 2021	Mandatory wearing of masks in addition to public enclosed spaces (including schools) in open public areas. Closure of restaurants after 10 p.m. and complete closure of restaurants in red regions Prohibition of the use of sports facilities Prohibition of gatherings in groups of more than 10 people and, from November 13, a ban on all socializing except for families Closure of gyms and sports clubs From October 17, schools were closed and distance learning was organized for primary and secondary school students Only sport activities of top athletes, individual athletes and sports with a maximum of 6 participants at a distance of 3 m were permitted Introduction of a curfew between 9 p.m. and 6 a.m. Restriction of movement to the municipality of residence
The third wave (spring 2021)	1^st^ April–12^th^ May 2021	Continuation of distance learning Shortening the curfew between 10 p.m. and 5 a.m. Opening of schools on April 12, 2021

∗School took place through remote (on-line) learning.

**Table 2:** Baseline demographic and anthropometric characteristics of the sample.

Grade	Generation (*N*)	Age (years)	Male sex (%)	Height (cm)	Weight (kg)	BMI (kg/m^2^)
6th grade	Prepandemic (*N* = 756)	11.3 ± 0.5	52.5	154.5 ± 7.5	48.6 ± 12	20.2 ± 3.9
	Pandemic (*N* = 853)	11.4 ± 0.5	51	154.2 ± 7.8	47.6 ± 11.8	19.9 ± 3.9
8th grade	Prepandemic (*N* = 756)	13.3 ± 0.5	52.4	166.4 ± 7.8	59.8 ± 13.1	21.6 ± 4
	Pandemic (*N* = 853)	13.4 ± 0.5	51	166.6 ± 8.3	60.3 ± 14.1	21.6 ± 4.3

**Table 3:** Absolute values of tests, analysis of variance, and difference between generations.

Test	Generation	6th grade	8th grade	*p*	Difference in 8th grade (95% CI)∗	Effect size (*η*^2^)
Tapping (*n*)	Prepandemic	37.8 ± 4.5	42.3 ± 4.8	0.619	0.2 (-0.6 to 0.1)	0.001
	Pandemic	37.4 ± 4.2	41.7 ± 4.8			
Standing long jump (cm)	Prepandemic	160.3 ± 24.2	179.9 ± 28.3	0.007	2.4 (0.6 to 4.2)	0.005
	Pandemic	162.3 ± 23.7	179.4 ± 29.9			
Polygon backwards (0.1 s)∗∗	Prepandemic	139.8 ± 42.5	122.8 ± 36.2	<0.001	7.2 (3.9 to 10.5)	0.013
	Pandemic	134.3 ± 40.0	126.4 ± 46.1			
Sit-ups (*n*)	Prepandemic	42.9 ± 9.4	46.9 ± 9.9	0.001	1.5 (0.7 to 2.3)	0.01
	Pandemic	42.5 ± 9.9	45.1 ± 10.5			
Stand and reach (cm)	Prepandemic	44.3 ± 8.4	47.2 ± 29.1	0.030	1.9 (-0.1 to 3.9)	0.002
	Pandemic	43.8 ± 8.4	44.1 ± 30.1			
Bent arm hang (s)	Prepandemic	46.8 ± 29.6	47.6 ± 29.7	0.006	1.8 (0.01 to 3.7)	0.003
	Pandemic	49.1 ± 29.8	46.9 ± 30.7			
60 m sprint (0.1 s)∗∗	Prepandemic	106.6 ± 11.5	99.3 ± 11.3	0.001	1.6 (0.7 to 2.5)	0.009
	Pandemic	106.6 ± 11.2	100.9 ± 12.6			
600 m run (s)	Prepandemic	163.1 ± 28.6	157.1 ± 30.7	<0.001	7.7 (5.0 to 10.4)	0.023
	Pandemic	162.7 ± 27.6	164.5 ± 34.8			
Physical fitness index	Prepandemic	50.4 ± 30.5	50.5 ± 29.7	<0.001	5.7 (3.8 to 7.6)	0.028
	Pandemic	51.6 ± 29.6	45.8 ± 30.3			

∗The adjusted average difference between generations in 8th grade is adjusted according to the baseline in 6th grade. ∗∗The result in polygon course backwards and in the 60 m sprint is measured in tenths of a second.

Abbreviations: ANOVA: Analysis of variance; CI: Confidence interval; *η*^2^: Partial eta squared.

**Table 4:** Demographic composition of 4 groups of students and body composition in 8th grade.

Generation	Municipality (*N*)	Male/female *N* (%)	Height (cm, male/female)	Weight (kg, male/female)	BMI (kg/m^2^, male/female)
Prepandemic	Urban (381)	200 (53.5)/181 (47.5)	168/164	61/58	21.4/21.5
	Rural (375)	197 (52.5)/178 (47.5)	167/164	61/59	21.5/21.8
Pandemic	Urban (430)	229 (53.3)/201 (46.7)	169/164	62/59	21.5/21.7
	Rural (423)	206 (48.7)/217 (51.3)	169/163	61/58	21.7/21.5

## Data Availability

The raw data including the anonymized test results is available from the authors upon request.

## Supplementary Materials

Supplementary Materials Supplementary Table 1: selected schools and municipalities.
