# Supplementary material for: Impact of the COVID-19 Pandemic on the Motor Development of Schoolchildren in Rural and Urban Environments
Source: Biomed Res Int. 2022 May 17;2022:8937693. doi: 10.1155/2022/8937693 (PMC9123420; doi:10.1155/2022/8937693)
Supplement: Supplementary Materials — Supplementary Table 1: selected schools and municipalities. [file 8937693.f1.docx]

Supplement Table 1

Table S1: Selected schools and municipalities

| **Municipality** | **Population density (n/km^2^)** | **School** | **Number of pupils (N)** | **Percentage (%)** |
| --- | --- | --- | --- | --- |
| **Municipalities with many**  **population density** | **499.3** | **/** | **811** | **50.4** |
| Ljubljana | 1068.3 | Vodmat | 74 | 4.6 |
| Maribor | 765.0 | Borcev za severno mejo | 59 | 3.7 |
| Izola/Isola | 586.5 | Dante Alighieri | 20 | 1.2 |
| Celje | 514.8 | Celje IV | 96 | 6 |
| Domžale | 509.1 | Venclja Perka | 96 | 6 |
| Piran/Pirano | 423.9 | Cirila Kosmača | 40 | 2.5 |
| Velenje | 401.6 | Antona Aškerca | 85 | 5.3 |
| Kranj | 376.3 | Franceta Prešerna | 133 | 8.3 |
| Murska Sobota | 288.1 | Murska Sobota III | 65 | 4 |
| Jesenice | 285.3 | Koroška Bela | 72 | 4.5 |
| Trbovlje | 273.9 | Ivana Cankarja | 71 | 4.4 |
| **Municipalities with small**  **population density** | **23** | **/** | **798** | **49.6** |
| Mislinja | 40.6 | Mislinja | 66 | 4.1 |
| Gornji Petrovci | 30.0 | Gornji Petrovci | 8 | 0.5 |
| Divača | 29.8 | Dr. Bogomirja Magajne | 53 | 3.3 |
| Žužemberk | 29.0 | Žužemberk | 88 | 5.5 |
| Pivka | 27.9 | Pivka | 78 | 4.8 |
| Gornji Grad | 27.7 | Frana Kocbeka | 32 | 2 |
| Semič | 26.5 | Belokranjskega odreda | 53 | 3.3 |
| Hrpelje - Kozina | 25.0 | Dragomirja Benčiča Brkina | 57 | 3.5 |
| Šalovci | 23.9 | Šalovci | 9 | 0.6 |
| Podvelka | 22.5 | Brezno Podvelka | 41 | 2.5 |
| Loška dolina | 22.2 | Heroja Janeza Hribarja | 69 | 4.3 |
| Kobarid | 21.1 | Simona Gregorčiča | 59 | 3.7 |
| Črna na Koroškem | 20.7 | Črna na Koroškem | 39 | 2.4 |
| Ribnica na Pohorju | 19.3 | Ribnica na Pohorju | 20 | 1.2 |
| Loški Potok | 13.4 | Loški potok | 29 | 1.8 |
| Luče | 13.4 | Blaža Arniča | 40 | 2.5 |
| Kostel | 12.3 | Fara | 11 | 0.7 |
| Bovec | 8.7 | Bovec | 46 | 2.9 |

Source: "Population Density and Femininity Index, Municipalities, Slovenia, Semiannual" (Table ID: 05C4010S), Statistical Office of the Republic of Slovenia, 2021.
